# An emergency department optimized protocol for qualitative research to investigate care seeking by patients with non‐urgent conditions

**DOI:** 10.1002/nop2.667

**Published:** 2020-10-23

**Authors:** Piers Truter, Dale Edgar, David Mountain, Caroline Bulsara

**Affiliations:** ^1^ School of Physiotherapy University of Notre Dame Australia Fremantle WA Australia; ^2^ Emergency Department Fiona Stanley Hospital Murdoch WA Australia; ^3^ SJOG Midland Public and Private Hospital Midland WA Australia; ^4^ Fiona Wood Foundation Perth WA Australia; ^5^ Emergency Department Sir Charles Gairdner Hospital Nedlands WA Australia; ^6^ University of Western Australia Nedlands WA Australia; ^7^ School of Nursing and Midwifery University of Notre Dame Australia Fremantle WA Australia

**Keywords:** access and evaluation, emergency service, healthcare quality, healthcare‐seeking behaviour, hospital; primary health care, qualitative research

## Abstract

**Aim:**

To describe a tailored qualitative research methodology for exploring the complex interaction of factors driving non‐urgent care seeking in the emergency department.

**Design:**

Qualitative descriptive design with a literature informed semi‐structured interview and analysis structure. Triangulation with the State‐Trait Anxiety Inventory allows expedited exploration of biopsychosocial factors. Consolidated criteria for reporting qualitative research requirements integrated.

**Methods:**

With a short 10‐ to 15‐min interview and a low‐inference analysis process, this methodology offers a structured way to explore the “go to ED” decision, to understand the patient perspective on their healthcare needs and feed into the development of suitable local services that meet patient healthcare needs.

**Results:**

This methodology offers a structured way for clinician–researchers to explore the factors that influence patients seeking care in the emergency departments for non‐urgent conditions that are specific to their local health service environment. The described methodology is accessible to novice qualitative researchers and includes the semi‐structured interview, coding and analysis frameworks.

## 1 INTRODUCTION

A proportion of patients seeking care in emergency departments (ED) have conditions that could potentially be better managed by community‐based healthcare services. There is debate about the proportion of ED attendances constituting this significant group, with estimates ranging from 10%–61% in Australia (AIHW, 2017; Nagree, et al., 2013). This group of patients are usually described from a health service perspective using labels such as “non‐urgent,” “low urgency,” “potentially avoidable general practice,” “GP (general practice) presentations” or “primary care” presentations. Yet one of the top reasons these patients seek care are their or significant others’ perceptions of needing urgent care (Lobachova et al., 2014; Masso et al., 2007; Shaw et al., 2013). The language used in the literature to characterize these patients demonstrates a potential systematic bias for health professional perceptions of the appropriateness of ED care seeking. Healthcare workers’ opinions about care‐seeking appropriateness have been explored (Breen & McCann, 2013) and are often dissonant to patient perspectives (Durand et al., 2012; Masso et al., 2007). To attempt to address this bias, we will define this group as patients with conditions with potential for self or supported management in the community (PSSM).

Developing ED avoidance strategies is often the goal of PSSM ED presentation research. Systematic health service perception bias may limit the usefulness of this research for health system redesign. Patients can be viewed as consumers of health services and potentially determine health system usage or design through the way they choose to seek care. Overall, ED may be an expensive way to deliver care for PSSMs with high prevalence conditions like low back pain (Edwards et al., 2017) and may not be very effective (Friedman et al., 2012; Machado et al., 2018). Using low back pain (LBP) as an example, many patients recover fully in short time periods and are effective in self‐management. Patients with LBP are however not a homogenous cohort and nuanced care may be needed by some patients (Maher et al., 2017). Rather than focusing on ED avoidance, possibly a more suitable health system goal would be partnering with patients, to develop care pathways suitable for diverse personal, clinical and societal needs and resources.

We posit that a rigorous qualitative methodology designed specifically for the ED clinical environment is needed to give voice to the patient perspective. This methodology requires several contextual factors embedded therein, including patient‐specific factors (Uscher‐Pines et al., 2013), condition‐specific factors, temporal factors and consideration of local health service options. Qualitative methodologies used to investigate care seeking in the ED have used grounded and narrative theory approaches which can take up to an hour in interview (Durand et al., 2012; Stafford et al., 2014). The challenge with qualitative research in the ED is to effectively explore the complex patient perspectives without interrupting patient care or imposing too great a research burden on patients with acute medical issues and in a clinical environment with limited time and space.

## 2 METHOD

### 2.1 Qualitative research framework

Due to the restrictions of patients on a rapid treatment pathway in modern ED services, we used a qualitative descriptive (QD) methodology because it is well suited to health service research and provides a robust but pragmatic process of interpretation and analysis. Sandelowski (2000) noted that the QD approach enables the researcher to “stay closer to their data and to the surface of words and events.” There is also less of a focus in the analysis phase when a descriptive method is employed (Colorafi & Evans, 2016). Sandelowski maintains the QD is “a comprehensive summary of events in every day terms of those events” (Sandelowski, 2000). QD is a suitable method in healthcare research as it helps to focus research questions directly on the experiences of the participants rather than through a more theoretical lens such as grounded theory or phenomenology (Neergaard et al., 2009). The practical application of this is that information provided by a patient in a healthcare setting can be taken verbatim and analysed with minimal further interpretation.

The QD methodology provides a framework for the interview, coding and analysis phases of the research. Firstly, during data collection, the QD methodology allows us to build on existing ED research by using a conceptual framework as a starting point. This is distinct from some other qualitative approaches that develop a framework through analysis being conducted in parallel with interviewing (data collection phase). A semi‐structured interview (refer to S1: Semi‐structured interview template) was developed directly from a theoretical model of PSSM care‐seeking decision‐making from an extensive systematic review (Uscher‐Pines et al., 2013). The semi‐structured interview simplifies the interview process because the interview questions are determined a priori. The structure is a sound base for maintaining inter‐ and intra‐interviewer reliability and makes this kind of research accessible to novice interviewers, as the interview has a clear trajectory from the start.

The conceptual framework also allows a structured approach to the coding phase of the QD process. The initial coding template (refer to S2: Coding template) was developed from the (Uscher‐Pines et al., 2013) model following QD analysis guidelines (Colorafi & Evans, 2016). Transcribed interviews are coded against this structure to develop themes; however, there is no requirement for researchers to rigidly adhere to the template. Rather the coding structure should evolve if new narratives emerge that do not fit in the existing framework.

The subsequent analysis phase follows QD principles, which is a low‐inference approach where the participants statements are taken verbatim and reported in everyday language (Sandelowski, 2000). This philosophical approach will also simplify analysis within the research team as it relies on the facts from the interview rather than interpretation of the interviews through a theoretical lens by the analysts.

### 2.2 The local environment

To validate our research methodology, participants will be recruited from a tertiary hospital ED that receives 110,000 presentations a year. This ED does not have a co‐located general practice and the catchment area is large, with significant variability in socio‐economic demographics (ABS, 2018) and potentially GP access. There is a private ED adjacent to the hospital and an urgent care centre within 10 kms. We will directly ask patients in interview why they did not choose these alternative services, testing their choice within the local healthcare options.

### 2.3 Population sample/setting

We hypothesize that the classification of patients as PSSM understates a considerable heterogeneity in presenting complaints and hence motivating factors to seek care. Careful consideration also needs to be given to recruitment about care seeking in the ED, as not all patients are responsible for that decision. We will use this methodology to investigate the care‐seeking behaviour of patients and provide example inclusion and exclusion criteria for one group we will investigate, namely adult patients with simple fractures.

#### 2.3.1 Inclusion criteria

The inclusion criteria will be English speaking, West Australian resident adults aged 18–65 (working age) self‐presenting to ED (i.e. not in police custody, from an aged care facility or a correctional facility), diagnosed with a simple closed fracture, presenting within 4 weeks of initial injury and triaged to Australasian Triage Scale Category 3–5.

#### 2.3.2 Exclusion criteria

Exclusion criteria will be minors, patients over 65, patients who cannot read or understand the study paperwork, patients with significant mental health issues, patients obviously affected by drugs or alcohol at the time of the interview, patients with open fractures or injuries likely to require surgery, any patient with medically diagnosed osteoporosis, diabetes with associated peripheral neuropathy or medically diagnosed abnormal bone structure.

### 2.4 Recruitment

Patients will be recruited from the ED waiting room while attending for treatment. They will be identified from the emergency department information system (EDIS) and approached by one of the project interviewers. Written consent will be obtained prior to interview.

A sampling matrix will be used to organize condition‐specific criteria that might influence patient care‐seeking behaviours and guide recruitment. An example sampling matrix is provided for our simple fracture study (refer to S3: Sampling matrix). In general, recruitment will be structured to ensure proportionate representation from office hours, evening and weekend presentations, as access to alternative care may vary. Sample size for the interview participants is to be determined through maximum variation sampling methodology (with reference to the sampling matrix) which is a non‐probability purposive sampling technique. Data saturation will be determined by agreement within the entire research team.

Basic demographic information (refer to S4: Demographic data structure) will be collected from each participant as per COREQ recommendation 16 (Tong et al., 2007) to give context to results.

### 2.5 Interview setting

Interviews will be conducted in a quiet private room adjacent to or within the ED. A quiet room is best for audio recording and reducing the stress of the usually noisy ED environment. Ideally, only the participant and the interviewer should be present, to reduce possible bias from family and friends being present. Where this is not possible, the presence of others should be noted for consideration in analysis. Data on the rate of participation will be kept for reporting per COREQ recommendation 13 (Tong et al., 2007).

### 2.6 Interviewers

There are some challenges in recruitment, specifically identifying suitable participants from triage information and fitting the interview into the patient care workflow. We have previously successfully engaged the assistance of experienced ED clinicians and external researchers in our studies. The latter require significant training and support to be effective in screening suitable participants. Good understanding of ED processes and workflow is also important to minimize participant distress and avoid patient care interruption. Using ED, clinicians is acknowledged as a potential source of bias, which we will address through engagement with hospital consumer advisory groups and role‐playing the interviews in a piloting process guided by an experience qualitative researcher. The reflexive training involves feedback to each interviewer on their interview technique and any potential or perceived bias.

### 2.7 Data collection methods

Participants will be recruited in the ED waiting room prior to treatment, as their perspective could change after their interaction with treating staff. Interviewers are present in the ED as researchers and not involved in patient care, which for clinicians is important from an ethical perspective. Participants first complete the STAI‐AD short‐form questionnaire (Spielberger et al.,^2015^). To get an accurate assessment of patient anxiety, it is ideal to complete the questionnaire prior to any reassurance from the treating team in the ED. A short 10–15 min semi‐structured interview (see S1: Semi‐structured interview template) completes the data collection. We will be scrupulous in trying to avoid interfering with a patient's care in the ED. Pragmatically we may have to conduct some interviews after the patient has been seen. These postcontact interviews will be noted, and the influence of interview timing will be considered in analysis. We will provide a verbal summary of the interview to each participant at the end of the interview to confirm our understanding and to allow them to correct any errors. Participant interviews will be recorded electronically using a single digital voice recording unit (Olympus WS‐853). All interviews will be recorded on this device, and the files will be transferred to a computer for transcription. Interviewers will record their perspectives on each interview on a standardized form (Refer to S5: Interview notes page [for interviewers]), after the interview to provide reflexivity to the data collected.

### 2.8 Anxiety in the ED

The design of the semi‐structured interview includes a short statement explaining the purpose of this study, namely to gain a patient perspective on care seeking. While we are trying to measure patient anxiety levels in relation to care seeking, the interview is designed to minimize patient distress. It is also noted that in addition to injury‐related concerns, there is often an anxiety amongst patients about the legitimacy of their need for emergency care (i.e. using the ED appropriately) (Coster et al., 2017). A previous qualitative study in a Western Australia ED (Abernethie & Nagree, 2004) discovered that this type of anxiety requires sensitive handling both to ethically engage with patients and to ensure an unrestricted narrative from the patient. That we are not challenging the legitimacy of the patient to seek care in the ED is directly addressed in the opening statement of the semi‐structured interview (refer to S1: Semi‐structured interview template).

### 2.9 Using an outcome measure to triangulate with the interview

The State‐Trait Anxiety Inventory for Adults (STAI‐AD) is a well‐established outcome measure consisting of two 20‐item questionnaires designed to measure participant anxiety (Spielberger et al., 2015). The first questionnaire (form Y‐1) measures state anxiety (participants current anxiety levels/arousal), and the second questionnaire (form Y‐2) measures trait anxiety (how likely the participant is to perceive a stressful situation as dangerous). With higher levels of trait anxiety, it is likely that a participant will have stronger state–anxiety reactions, that is they are generally more anxious. This questionnaire was used to measure the anxiety in participants in relation to their injury and will be referred to in the interview (triangulation) to try to determine how influential anxiety was in seeking care in the ED.

The STAI‐AD has been validated and standardized (Spielberger et al., 2015) and has been used extensively in research on condition related anxiety in patients seeking medical care. The full STAI‐AD takes 10 min to administer; however, there is a validated 20 question short form (Spielberger et al., 2015), which has less participant burden. We will use the short form as it seems more setting appropriate and timely.

### 2.10 Data analysis

A deductive approach will be taken with coding the interview transcripts and the initial coding structure is based on the Uscher‐Pines et al. (2013) model (refer to S2: Coding Template). We use QSR NVivo (Version 12) for data management during the analysis process. Validity of the coding process and identification of themes will be achieved by the independent coding of 2–3 cases by two of the research team. This will then be discussed with the entire research team to achieve consensus on coding practice. Once the coding structure is agreed, the remaining transcript coding will be conducted by a single researcher. It is expected that the coding structure may evolve during the coding process following template analysis principles (Brooks & King, 2012) if new themes emerge. The research team will also consider whether the timing of the interview (before or after care) has affected participants providing accurate explanations as to why they sought care in the ED.

Directed content analysis (Colorafi & Evans, 2016), using the Uscher‐Pines et al. (2013) model as a theoretical framework, is then used to identify themes in parallel by two separate researchers. Their analysis will be presented and discussed by the entire research group for agreement.

### 2.11 Rigour

Rigour in qualitative studies is evaluated through the criteria of credibility, dependability, confirmability, transferability and authenticity (Colorafi & Evans, 2016; Cope, 2014). These are described in Table 1 below showing how they apply to our methodology.

**Table 1:** Trustworthiness criteria for qualitative research and application of these in this methodology

Trustworthiness criteria	Application to this study
*Credibility* Truth in representation and interpretation of participant views.	Seek “negative cases” for hypotheses Test rival explanations Seek explanation for inconsistencies discovered in triangulation processes (Tolley et al., 2016) Reporting on researcher experience Report on participant engagement Report on interview process Maintain an auditable research process
*Dependability* Consistency of the data over similar conditions	Collaborative and parallel decision‐making with audit by multiple researchers Actively manage individual researcher bias
*Confirmability* Ability to demonstrate that the data represent participant viewpoints and not pre‐existing researcher bias(es).	Description of process for interpretation of data Demonstrate themes in data with direct rich quotation in reporting Maintain an auditable research process
*Transferability* Findings can be generalized and applied to other similar contexts	In reporting, provide sufficient information on participants and the research context to allow readers to evaluate transferability
*Authenticity* The extent of faithful expression of participants feelings and emotions	Reporting allows readers to understand the participants experience through direct quotation.

We developed this methodology using the consolidated criteria for reporting qualitative research (COREQ) guidelines (Tong et al., 2007). Bias minimization strategies will include using interviewers with experience of the ED environment, training the interviewers (involving reflexive training (Richards & Emslie, 2000)), practising the interview and providing strategies for difficult interviews (Tolley et al., 2016)), using interviewers who do not have a clinical relationship with the participants, consistent disclosure of interviewer/ study motivation and project oversight by an experienced qualitative methods researcher. These strategies will be reported along with simple demographics on the interviewers. The semi‐structured interview will also help to reduce interviewer bias as it includes a consistent scripted introduction, confidentiality statement and description of the flow of the session for each interview (Ranney et al., 2015).

### 2.12 Ethical considerations

There are three main ethical issues associated with interviewing patients attending ED. The primary issue is to ensure that participation in the qualitative research process does not interfere with their treatment. We aim to interview patients before they are assessed and treated to understand their decision‐making process, without risk of bias from interaction with ED staff. We however recommend in the patient's best interests that the interview occurs pragmatically when patients are waiting, even if this happens after they have been treated. The second issue relates to patients becoming distressed during the interview, potentially because of a painful condition. Again, in this case patient care should be prioritized. We recommend ceasing the interview and alerting the ED staff to the distressed patient. EDs typically have well established multi‐disciplinary approaches to managing a patient distress and the patient should be connected to these processes. Finally, we recommend taking an active approach to affirming the patient's rights in choosing to attend ED. A proportion of patients may worry about the appropriateness of choosing to seek care in the ED, and we recommend scripting an introduction in the interview to address this issue (refer to S1: Semi‐structured interview template). This research methodology has ethical approval through the South Metropolitan Health Service HREC (RGS0000001423).

## 3 DISCUSSION

Surveys, retrospective audits and epidemiological study designs are useful for identifying patterns in health service usage, local health service contextual factors and specific patient cohorts that might be relevant to health service design. Surveys can identify potential issues, for instance 37% of non‐urgent patients in an Australian ED, identify ED as the most suitable for their condition to be treated (Unwin et al., 2016). Qualitative research methodologies however provide insight and interpretation of those patterns vital to developing health services to match consumer needs. For instance, from a survey Unwin et al. (2016) found 40% of patients attend ED seeking tests (e.g. X‐ray). Interviewing patients seeking testing, Durand et al. (2012) identified that many patients understand the health system and chose to seek care in the ED because it was the most convenient option. They identify ED as a place with co‐located expertise, treatment and investigation services (e.g. radiology, pathology). Another example is that, lack of access to appointments with GPs and perceived urgency are often cited as a reasons to attend an ED. Qualitative research differentiates the respondents into a worried group of patients seeking urgent care (where they cannot access a timely appointment) and another group of patients who continue to work and who seek care at a time that does not interrupt their working day (Durand et al., 2011).

We combined a QD methodology (Sandelowski, 2000) with semi‐structured interviewing technique to place the patient perspective at the centre of the research. The semi‐structured interview technique is based on a conceptual model of patients with conditions with potential for self or supported management in the community (PSSM) decision‐making around care seeking (Uscher‐Pines et al., 2013) which allows exploration of ideas and concepts while ensuring that the research questions have been addressed. The semi‐structured interview approach facilitates a short focused 10‐ to 15‐min interview. Other more theoretically driven methodologies such as narrative or grounded theory have been used in ED, but require 10‐ to 75‐min interviews (Durand et al., 2012; Stafford et al., 2014). This shorter and more intensive approach is ideal for use in the ED, which has constraints on available space for interviews, performance indicators around patient's length of stay and where it is important to avoid interruption of patient care (Ranney et al., 2015). This approach also imposes a low burden on the patient who may be in pain or unwell.

Qualitative research also puts the patient perspective at the forefront. Often research investigating the care‐seeking behaviours of PSSMs has been based on a health system‐focused theoretical framework that describes care seeking by this group of patients as inappropriate or avoidable (McHale et al., 2013; Morris et al., 2018). The theoretical position is that PSSMs have conditions that can be managed in ED, but equally could be managed effectively and perhaps better by community services. There is however considerable variation in the definition of PPSMs, with authors using urgency (Unwin et al., 2016), general practitioner assessment of patients in ED (Whyatt et al., 2019), treatment by advice alone (McHale et al., 2013; Morris et al., 2018) and ED physician opinion (Morris et al., 2018). These theoretical frameworks have several problems which may limit their usefulness as a basis for research and in informing health system design to meet the needs of consumers.

The first issue is that patients are consumers of health services and make their care‐seeking decisions based on their available health service choices, their perception of the suitability of these services for their condition and the costs to them for these services. The availability of other services is variable, with many services closed on weekends and offering appointments around business hours (0800–1700). Patients may choose different health services on weekends, in business hours and after hours, depending on their available choices (Abernethie & Nagree, 2004). It should be noted that some patients are sophisticated health service users and exhibit pragmatic consumer behaviour for seeking care in the ED (Durand et al., 2012). They also may understand the health services in their area and appropriately choose the ED for treatment of a minor condition (Sprivulis, 2003).

The relationship of other health services to ED is also potentially complex. Some patients seem to attend ED because they cannot access a primary care provider (Penson et al., 2012; Uscher‐Pines et al., 2013), while others avoid primary care because they feel that their condition needs specific ED treatment (Penson et al., 2012). Other patients are unable to attend primary care during business hours and attend ED because they are unwilling to take time off work to seek care (Durand et al., 2012). A proportion of non‐urgent patients are also referred to the ED by their primary care physicians (Unwin et al., 2016). Understanding the specific local issues facing ED is however vital to developing effective alternative treatment pathways. From an academic perspective, the decision to go to ED is always made within a local health service landscape, potentially limiting the generalizability of research from other health service contexts.

Additionally, the decision to go to ED is often complex and specific to the personal situation and history of each individual patient. This decision‐making process includes cultural, social, financial and temporal factors. It is influenced by individual healthcare beliefs, perceptions of severity and the emotional state of the patient. Anxiety and distress linked to a condition (Koziol‐McLain et al., 2000) and pain from an injury are important factors for seeking care in the ED (Abernethie & Nagree, 2004; Coster et al., 2017), even in patients who acknowledge that they do not believe they have a serious injury (Durand et al., 2012). In our methodology, we include the STAI questionnaire to allow a time‐efficient process for understanding the emotional state of the patient and its relevance in care‐seeking decision‐making.

In many cases, acuity and urgency are used to define “appropriate care seeking” in the ED (Durand et al., 2012) and this may not be a relevant classification approach to direct research. There is almost certainly significant heterogeneity of conditions within the PSSM group, and this presents an analytical problem for researchers using this definition (Van den Heede & Van de Voorde, 2016). Care‐seeking behaviours may be very different for individuals with different conditions. It is very likely that care seeking in the ED by PSSMs may be linked to their presenting complaint. For example, a pregnant lady with haemoptysis (diagnosis in ED: gastroesophageal reflux) arrived by ambulance at the onset of symptoms, while a patient with dental pain (diagnosis in ED: toothache) attended ED after nine months of pain (Koziol‐McLain et al., 2000).

Defining non‐urgent patients by their triage category (i.e. acuity) can also hide the potential complexity of patient care needs. Mazza et al. (2018) investigated “potentially avoidable GP presentations” to ED by older (>70) patients and defined their study cohort by age and acuity (Australasian Triage Scale 4 and 5). Australian EDs often have dedicated multi‐disciplinary teams for patients over 65, as they are at risk of fall and often have more complex needs for safe discharge (Nagree et al., 2013). Many older patients are triaged to low acuity categories and may be considered PSSMs, but may benefit from the multi‐disciplinary assessment and treatment available in the ED.

Another underlying assumption is that EDs have the clinical capacity to optimally treat the diverse range of conditions afflicting PSSMs. In the case of musculoskeletal conditions, for example, there is some evidence that this may not the case for ankle sprains (Konradsen et al., 2002) and LBP (Kamper et al., 2020). ED is a clinical environment where there is typically a single patient contact and then on‐referral. This contact is often focused on acute management and screening patients for referral to hospital services. Rather than ED avoidance being the goal of research, the issue of appropriateness might be helping patients to find the support that they need individually from health services better equipped to treat their condition.

Overall, each patient weighs up the merits of seeking care in the various local health services including factors such as previous experiences of care, cost of care and the patient's perception of the appropriateness seeking care in the available services (e.g. that a service is equipped to treat their condition).

We propose that the qualitative methodology we describe allows effective consideration of the complexities of consumer healthcare‐seeking behaviour. The semi‐structured interview allows time‐efficient patient engagement. Flexible coding against a coding framework linked to the interview allows patient narratives that are not initially present to emerge. The underlying QD process should allow this methodology to be easily adapted to local research. We will use this methodology to investigate several different PPSM sub‐groups including simple fractures, LBP, ankle sprains and sports injuries.

### 3.1 Limitations

The main limitation to our qualitative research methodology relates to achieving a full interview without interrupting patient care in the ED. It is ideal to conduct the interview with the patient alone, in a quiet room, prior to treatment starting the ED. The reality of a busy ED is that it is likely that some or all of the above may have to be pragmatically compromised to avoid interrupting patient care. We recommend taking careful notes on the deviations from the ideal protocol and considering these during analysis.

Another limitation is that the results of a study conducted following our protocol will produce results that are highly specific to the local area of the study. While this may be useful to feed into local healthcare system redesign, careful consideration needs to be given to the generalizability of the results to other settings. Finally, the semi‐structure interview and coding template facilitate qualitative research in the ED by researchers with minimal experience. Care must be taken however in the interviewing and coding phases to allow new relevant themes to emerge. Interviewers should not stick rigidly to the interview questions and should explore new themes with follow up questions. The semi‐structured interview should give structure to the interview that allows relevant information to come to light. Similarly, in the coding process, if a theme does not fit into the coding structure, that structure should evolve to include new themes/ sub‐themes. A rigid adherence to the interview/ coding structures may bias the results.

## 4 CONCLUSION

This qualitative research model will integrate primary decision‐making factors, acknowledges that social and personal context influence the decision and places the “go to ED” decision within a spectrum of possible care‐seeking options. It offers a structured way to explore the decision and the potential for targeted interventions to change that decision (i.e. seek care in an alternative service).

## CONFLICT OF INTEREST

The authors have no conflicts of interest.

## Supporting information

Supplementary Material

## Data Availability

As this is a protocol, there are no participant data. However, to support use of this protocol by other researchers we are providing five supplementary documents which are referenced in text.
