# Supplementary material for: An emergency department optimized protocol for qualitative research to investigate care seeking by patients with non‐urgent conditions
Source: Nurs Open. 2020 Oct 23;8(2):628–35. doi: 10.1002/nop2.667 (PMC7877135; doi:10.1002/nop2.667)
Supplement: Supplementary file 1 — Supplementary Material [file NOP2-8-628-s001.docx]

## S1: Semi-structured interview template

**Patient motivation for seeking care in the ED with a simple fracture**

**INTRODUCTION:***Interviewer to wear a name badge identifying them as a hospital employee.*

*Interviewer to introduce themselves by name and as a researcher (who works in the ED) rather than as a clinician. We are aiming to maintain a researcher to participant relationship rather than a clinician to patient relationship. The interviewer must remain distant from the clinical interaction in the ED.*

*Can you please fill in this short questionnaire? The questionnaire is to help us understand how worried you are about this injury.* **Interviewer to provided participant with STAI on a clipboard.**

*To start the interview - read word for word*

Thank you for agreeing to be interviewed. Prior to starting I want to let you know that the recording of this interview will be transcribed and then de-identified. We will remove your name and any identifying personal details from the transcription. This means that your comments will be anonymous.

The aim of this study is to help develop emergency department services that match the needs of patients. We would like to understand your reasons for coming to the ED so that we can help others in the future.

We are investigating the reasons why patients come to the emergency department. We know that everybody comes to the emergency department because they are concerned that they have an injury that needs investigating by a health professional. If you have made the decision that you need the services of the ED, then we respect your decision. In this study we are trying to understand how you made the decision, as well as your reasons for coming and what services you were seeking from the emergency department.

We are interested in what prompted you to come to the emergency department. We are not asking about the detail of your injury, but rather what you hoped to get from emergency department (services / outcomes).

Please let me know if you need to stop or if you become uncomfortable.

*Interviewer notes: It is very important that patients understand that we are not challenging the appropriateness of their decision to seek care in the ED. This must be clarified before moving on.*

**Demographic questions:**

*Interviewer notes: These are both ice-breaker questions and the information is used in evaluating the impact of the injury on the participant.*

- Occupation
- Dominant hand (if upper limb injury)

**Theme : Advice or referral**

*Interviewer notes: Referral from a Primary care practitioner (PCP) is thought be a very influential reason for seeking care in the ED. It is also hypothesised that relatives, friends and partners are also important motivators for seeking care in the ED.*

**QUESTION:** Have you seen anybody about your injury (e.g doctor, physiotherapist) before coming to the ED today?

**PROBE Q:** Did anybody else suggest you come to the emergency department?

**PROBE Q:** In terms of deciding to come to the emergency department; how important was the advice you received in that decision?

**Theme: Perceived severity**

*Interviewer notes: There are two sub-themes in this section. The first is that the patient has personal beliefs / knowledge of the injury that suggests that it needs urgent care. The second is that they are worried / anxious about the injury and this raises the urgency of care seeking. The STAI questionnaire will provide information on how anxious the patient is about their injury (now) and how anxious they are in general. We are aiming to establish if this worry / anxiety made care seeking urgent.*

**QUESTION:** Thinking back to making the decision to come to ED; how quickly did you think your injury required treatment?

**PROBE** Within a couple of hours, within 12 hours, within 24 hours?

**QUESTION:** Interviewer to look at STAI form and triangulate response on that form with the participant, e.g. The survey that you completed before the interview suggests that understandably you were / were not worried about this injury. Is there anything in particular that is making you worried about your injury today?

**Theme: Convenience**

*Interviewer notes: This theme relates to the ease with which a patient can seek care including travel, timing and location. The idea that ED is a ‘one-stop-shop’ compared to multiple appointments with their GP / radiology / etc. This is an important driver of ED usage in the literature.*

**QUESTION:** I’d like to talk to you a bit about the actual experience of accessing the ED. Thinking about the time of your injury again, how convenient was it to have your injury seen in the ED?

**PROBE Q:** How does this compare to alternatives to ED?

**PROBE Q:** How could ED be made more convenient? Or if the experience was good, what was it about the ED that made it convenient for you?

**Theme: Beliefs / knowledge about alternatives**

*Interviewer notes: There are several ideas to test in this section. The first is whether the person believes that they have an injury that can only be managed in ED. The second is whether they feel that ED care for this injury will be superior to the alternatives. Finally, some patients may be seeking a ‘second opinion’ as they were not satisfied with the first opinion they received.*

**QUESTION:** Thinking back to the time of your injury, can you talk a little about why you chose to get treatment in the emergency department?

**PROBE Q:** Were there any other health services (e.g. GP, physio ) that you would have considered? injury?

**PROBE Q:** What facilities and services does the ED have that the other providers don’t?

**Theme: Access / availability**

*Interviewer notes: The issues in this section are patients not having a regular doctor / physio and having difficulty accessing alternative care. Access issues include being unable to get an urgent / timely appointment, injuries occurring out of office hours (and hence restricting access to alternative care) and busy professionals being unwilling to take time off work to seek care in office hours (so they come to ED out of hours).*

**QUESTION:** Thinking back to the time of your injury, did you have any difficulty accessing health care services outside the emergency department?

**PROBE Q:** Did difficulty accessing alternative healthcare services influence your decision to come to the emergency department? **[Follow on]** Do you think your injury could have waited for a routine GP appointment?

**PROBE Q:** *(For in office hours interviews, where the injury occurred out of hours or on a previous day)* I notice that you were injured [at time / date x], but you came today in office hours. Can you tell me why you did not come to the emergency department at the time of injury?

**PROBE Q:** Do you have a regular GP?

**PROBE Q:** Do you have a regular physiotherapist?

**Theme: Cost**

*Interviewer notes: The primary issue here is cost of care, however continuing from the last section, there might be an opportunity cost for care seeking (leave / lost wages) that affect patients. Cost of care can include medical costs, but also costs from investigations and treatment.*

**QUESTION:** When choosing to come to the emergency department, did you consider the cost of ED versus the alternatives? Please explain.

**PROBE Q:** What costs were you worried about? – eg. Equipment, medication, bracing, appointment costs etc.

**CLOSING**

**Summary**

*Interviewer to provide a brief summary of the conversation, allowing the participants to clarify or add information.*

*You will probably be asked some of these questions again by the person treating you in ED, they will not have access to your replies in this interview (as it is anonymous).*

*Thank you for participating in our study. If you are interested in finding out the results please give me an e-mail address and I will send them to you when they are published.*

Interviewer to record their impressions after each interview

## Scripted answers to specific questions

Please defer answering these questions until after the interview is concluded.

**What did the questionnaire tell you?**

The Stait trait anxiety index (STAI) is used to measure how worried people are about their injury or health condition. In your case the results suggest that you are / are not worried about this injury.

**Why are you doing this study?**

This study is part of a group of studies that are looking at how simple fractures are managed from WA emergency departments. The overall aim is to redesign how people access care to make it more convenient and to hopefully reduce the cost for patients and the health system.

Our first study looks at how well people have healed from their injury after being seen in the ED. This is our second study and we are aiming to make sure that we have a clear understanding of the issues in seeking health care from our patients. Finally, we are going to survey general practitioners to find out if they have any issues with managing these fractures. When we have all this information, we are going to use it to redesign the system.

## S2: Coding Template

| Advice or referral (Part of the decision to seek care in the emergency department was ^advice or referral from^ | |
| --- | --- |
| *A primary care practitioner*   - A health professional provided advice to the participant that influenced their decision to attend ED   *A friend, relative or partner*   - A friend, relative or partner provided advice to the participant that influenced their decision to attend ED | *Referral from a Primary care practitioner (PCP) is thought be a very influential reason for seeking care in the ED. It is also hypothesised that relatives, friends and partners are also important motivators for seeking care in the ED.* |

| Perceived severity (The participant decided that they had a significant injury that required urgent attention ^because of^ | |
| --- | --- |
| *Knowledge about the injury*   - Based on their knowledge of the injury, the patient felt they had a significant injury that required urgent ED care.   *Personal beliefs about the injury*   - The participant believed (read as there is a societal narrative) that they had an injury that required urgent ED care   *Worry / anxiety about the injury.*   - The participant became anxious about the severity of their injury and sought urgent ED care as a result. This can include patients who are naturally anxious or heightened anxiety due to pain and injury. | *There are two sub-themes in this section. The first is that the patient has personal beliefs / knowledge of the injury that suggests that it needs urgent care. The second is that they are worried / anxious about the injury and this raises the urgency of care seeking. The STAI questionnaire will provide information on how anxious the patient is about their injury (now) and how anxious they are in general. We are aiming to establish if this worry / anxiety made care seeking urgent.* |

| Convenience (A consideration in attending the emergency department was reducing the logistical burden of care seeking ^in regards to^ | |
| --- | --- |
| *Reducing the amount of travel to health care services*   - The participant identifies that attending the ED is convenient as it will reduce the amount of travel involved in accessing health care   *Being time efficient*   - The participant felt that accessing care in the ED would take less time than the alternatives   *Being in a convenient geographic location*   - The emergency department is conveniently located   *Being a ‘one-stop-shop’ for management of this injury*   - The participant identifies that it is possible to access expert opinion, radiology, medication and other services in the ED. This contrasts to having multiple appointments in multiple locations if they sought care in alternative services. | *This theme relates to the ease with which a patient can seek care including travel, timing and location. The idea that ED is a ‘one-stop-shop’ compared to multiple appointments with their GP / radiology / etc. This is an important driver of ED usage in the literature.* |

| Beliefs / knowledge about alternatives (The emergency department was selected by the participant as the most suitable place to have their injury managed ^as a result of^ | |
| --- | --- |
| *Believing that this type of injury can only be managed in an emergency department*   - The participant believes that there are no suitable alternatives to treatment in the ED   *Believing that emergency department management of this type of injury is superior to the alternatives*   - The participant identifies that this type of injury could be managed by alternative services, but has decided that the best possible care is accessed in the ED   *Lacking faith in information / diagnosis / treatment initiated for this injury in an alternative service prior to seeking care in ED*   - The participant is attending ED seeking a second opinion on the management / diagnosis of their injury, as they are not satisfied with previous care | *There are several ideas to test in this section. The first is whether the person believes that they have an injury that can only be managed in ED. The second is whether they feel that ED care for this injury will be superior to the alternatives. Finally, some patients may be seeking a ‘second opinion’ as they were not satisfied with the first opinion they received.* |

| Access / availability (The decision to seek care in the ED included issues ^of access to^ | |
| --- | --- |
| *A regular doctor or physiotherapist*   - The participant does not have a ‘usual’ primary care provider. This may be by choice, or because there are difficulties gaining an appointment with a consistent clinician.   *An urgent or timely appointment*   - The participant was unable to book an appointment in primary care within a time frame that they deemed suitable for managing this injury.   *A primary care appointment out of hours*   - The injury occurred out of normal office hours and the participant sought care in the ED by default as there were few alternatives.   *A primary care appointment that suited their work schedule*   - The participant came to ED by choice as they were unwilling to take time off work to access an appointment in primary care | *The issues in this section are patients not having a regular doctor / physio and having difficulty accessing alternative care. Access issues include being unable to get an urgent / timely appointment, injuries occurring out of office hours (and hence restricting access to alternative care) and busy professionals being unwilling to take time off work to seek care in office hours (so they come to ED out of hours).* |

| Cost (The decision to access care in the ED included financial considerations) ^of the cost of^ | |
| --- | --- |
| *Private health care services*   - Incurring the cost of GP, physio and other healthcare services was considered when choosing to attend ED   *Radiology services*   - Incurring the cost of radiology services including x-ray, ultrasound, CT scan and MRI was considered when choosing to attend ED   *Orthopaedic support*   - The participant decided that they needed a brace, plaster or other support and incurring the cost of this equipment was considered when choosing to attend ED   *Mobility aid*   - The participant decided that they needed a walking aid and incurring the cost of this equipment was considered when choosing to attend ED   *Opportunity costs*   - The participant was unwilling to lose wages or use leave to seek care in office hours | *The primary issue here is cost of care, however continuing from the last section, there might be an opportunity cost for care seeking (leave / lost wages) that affect patients. Cost of care can include medical costs, but also costs from investigations and treatment.* |

## S3: Example sampling matrix

|  | **18-30** | **30-50** | **51-65** |  |  |
| --- | --- | --- | --- | --- | --- |
| **Age** |  |  |  |  |  |
|  | **Male** | **Female** |  |  |  |
| **Gender** |  |  |  |  |  |
|  | **Office hours (Week day)** | **After hours (Week day)** | **Weekend** |  |  |
| **Time of day** |  |  |  |  |  |
|  | **Toe** | **Clavicle** | **5^th^ metatarsal** | **5^th^ metacarpal** | **Radial head** |
| **Fracture type** |  |  |  |  |  |

Initially the interviewers will seek a convenience sample of participants. The sampling matrix will allow the team to identify patient groups not represented in the sample and to target them for recruitment.

## S4: Demographic data structure

This is the demographic data that we will collect from participants from the emergency department information system (EDIS) and the medical record. This data helps with the sampling strategy (as we are aiming to sample based on demographics) and in analysis of the interview.

Information that we will collect is;

- Age
- Gender
- Time of day of ED visit
- Fracture type
- Dominant hand
- Occupation
- Referral method to ED (self / GP / physio / relative / healthdirect /other)
- Postcode
- Previous ED usage (ED information systems)

## S5: Post-interview reflection (for interviewers)

“It is often beneficial to have a debriefing after the participants leave, but before the research staff departs a session. This allows the interviewer to record initial impressions while the verbal and non-verbal data are fresh in memory, clarify any ambiguous data that were collected and record any pertinent details of the session that may not be captured in the notes or recordings.” (Ranney et al., 2015)

| **Interview ID number** |  |
| --- | --- |
| **Impression of the key issues causing this participant to attend ED** |  |
| **Was this patient defensive or providing answers that might not reflect their true reasons for attending ED? Explain** |  |
| **Describe any other issues from this interview** |  |
